# Immune Activation Effects at Different Irradiated Sites and Optimal Timing of Radioimmunotherapy in Patients with Extensive-Stage Small Cell Lung Cancer: a Real-World Analysis

**DOI:** 10.1186/s12575-023-00217-y

**Published:** 2023-09-14

**Authors:** Min Wu, Shihao Wu, Yuetong Chen, Liangchao Sun, Jundong Zhou

**Affiliations:** 1 Department of Radiation Oncology, Nanjing Medical University, Nanjing, Jiangsu China; 2 Suzhou Cancer Center Core Laboratory, The Affiliated Suzhou Hospital of Nanjing Medical University, Suzhou, Jiangsu China; 3 Department of Radiation Oncology, Shandong Cancer Hospital and Institute, Shandong First Medical University and Shandong Academy of Medical Sciences, Jinan, China; 4 Medical School, Anhui University of Science and Technology, Huainan, China

**Keywords:** ES-SCLC, Radioimmunotherapy, Irradiated Organs, Inflammatory Blood Indexes, Interposed Timing

## Abstract

**Background:**

In view of the limited data on radiotherapy (RT) combined with immunotherapy in patients with extensive-stage small cell lung cancer (ES-SCLC), this study aimed to identify the immune activation effect on different sites and the survival outcomes of radioimmunotherapy at different treatment stages.

**Methods:**

Forty-five patients diagnosed with ES-SCLC were included in this retrospective analysis. We collected the overall survival (OS) of the patients,, recorded the blood cell counts before, during, and after RT, and derived blood index ratios such as the neutrophil-to-lymphocyte ratio (NLR), platelet-to-lymphocyte ratio (PLR), and systemic immune-inflammation index (SII). The datasets were analyzed using the Spearman rank correlation test, Kruskal–Wallis rank sum test and logistic regression.

**Results:**

Among the selected blood indices, the delta-NLR/PLR/Sll correlated with different irradiated organs, and the mean ranks of these three indices were the lowest in the brain-irradiated group during immunotherapy. Additionally, adjunct first-line immunotherapy with RT demonstrated a significant improvement compared to second- or third-line therapy and subsequent therapies.

**Conclusion:**

Our findings suggest that compared to other organs, the strongest immune activation effect occurs with brain RT, and ES-SCLC patients who received radioimmunotherapy (RIT) earlier achieved higher OS rates.

## Introduction

According to the American Cancer Society, lung cancer is estimated to be the leading cause of cancer-related deaths, with 609,360 deaths reported in 2022 [1]. Unlike non-small cell lung cancer (NSCLC), small cell lung cancer (SCLC) represents 15–20% of lung cancer cases with aggressive tumor characteristics, including a high growing rate, strong predilection for early metastasis, and poor prognosis. Consequently, the five-year-survival rate is less than 7% [2]. More than two-thirds of patients are initially diagnosed with ES-SCLC, with a 5-year overall survival (OS) rate of no more than 2% [3, 4]. After more than two decades of no clinical progress, platinum-based chemotherapy (CHT) has been replaced by a combination of CHT and immune checkpoint inhibitors (ICIs). This combination has been shown to prolong the OS and has been approved as the first- and third-line therapeutic regimen for ES-SCLC [5–7]. However, therapeutic resistance is inevitable in some patients, underscoring the need to develop more effective adjuvant therapies, such as local radiotherapy (RT), to enhance the efficiency of ICIs [8, 9].

RT is a major therapeutic strategy for various cancers. When combined with programmed cell death receptor-1(PD-1)/programmed cell death receptor-ligand 1 (PD-L1) signaling blockade, RT has shown significant clinical benefits in terms of survival outcomes [10]. RT activates the immune system by enhancing antigen presentation and promoting the infiltration of inflammatory cells [11]. Several studies have shown that inflammatory cells, including neutrophils, macrophages, and lymphocytes that interact with tumor cells, are the major modes of immunotherapy [12, 13]. Because of the significant role of systemic inflammation in tumor promotion and progression, the impact of different systemic inflammation markers are associated with patient outcomes in multiple solid neoplasms, including ES-SCLC. Studies have identified NLR, PLR and SII as predictive biomarkers of response to immunotherapy in SCLC [14, 15]. Therefore, considering the limitations of retrospective clinical studies on SCLC, we collected the inflammatory indicators to indirectly reflect the activation of immune system activation during RIT [16].

Studies have documented the major metastatic localizations in patients with lung cancer, including the brain (15%–43%), bone (19–33%), liver (33–40%), adrenal glands (18–38%), and kidneys (16–23%) [17]. Despite systemic immunotherapy, local radiation is the most common and effective treatment for these metastases [18]. Genetic heterogeneity between primary lesions and metastatic organs contributes to variations in immunogenic cell death and persistent antitumor immune activity at different irradiated sites [19]. Both stereotactic radiosurgery (SRS) and whole-brain radiotherapy (WBRT) showed improved time to central nervous system progression in ES-SCLC [20]. The most common site of bone metastases is the spine (64.7%) and local control rates of up to 95% can be achieved by RT [21, 22]. A meta-analysis showed that chemoradiotherapy resulted in a 48% reduction in thoracic progression as the first site of failure [23]. Radiation therapy for other metastases, such as liver and adrenal gland metastases, has also demonstrated great benefits. However, given the intratumor heterogeneity within SCLC subtypes [24], the combination of immunotherapy and RT may exhibit significant differences in efficacy when targeting different metastases. Therefore, it is important to explore the differences among the irradiated organs during RIT in patients with ES-SCLC [25].

In this study, blood indicators from patients undergoing RT for brain, bone, and lung metastases were collected, and changes in blood derived NLR, PLR, and SII ratios were analyzed to evaluate the activation effect of the immune system during RIT. By assessing patient survival outcomes, we summarized the influence of different treatment stages on the effect of RIT. The results of this study are expected to aid in the selection of irradiated sites and appropriate timing for combining immunotherapy with RT for patients with ES-SCLC.

## Methods

### Patient Selection

**Fig. 1:**
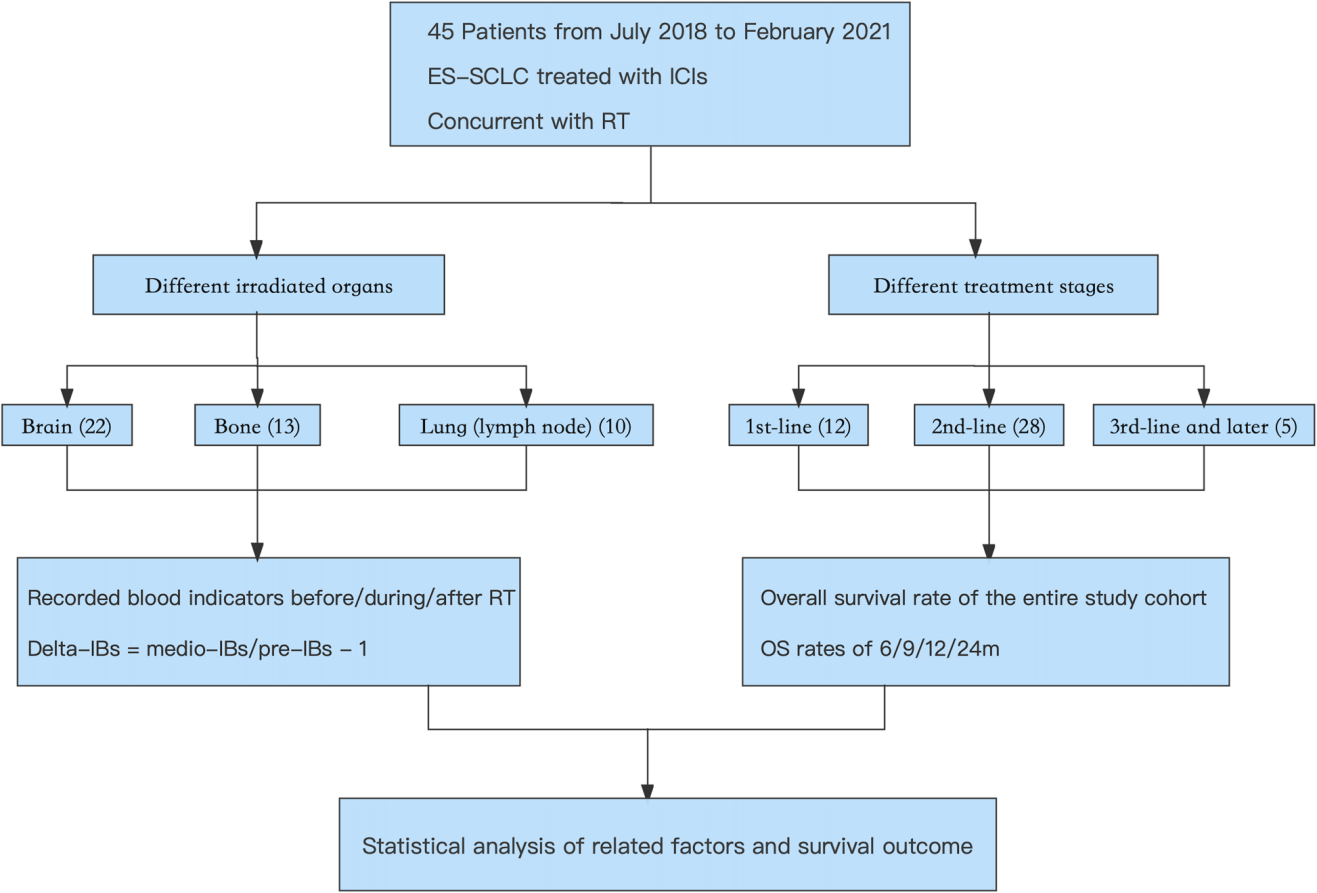
Flow Chart of the Study Design

This real-world analysis was aimed to identify which organ irradiated during immunotherapy has the strongest immune activation effect and outcome in ES-SCLC patients (Fig. 1). We included 45 cases who underwent irradiation to different metastatic organs or a primary tumor during their immunotherapy in Shandong Cancer Hospital, diagnosed from July 2018 to February 2021. These patients had received immune monotherapy or a combination of vascular endothelial growth factor receptor (VEGFR) therapy as well as chemotherapy. The inclusion criteria encompassed: (1) age; (2) Eastern Cooperative Oncology Group (ECOG) performance status; (3) therapy modes; (4) irradiated sites; (5) the timing of RT. Besides, the exclusion criteria encompassed: (1) age lower than 18-year-old; (2) the score of ECOG larger than 3; (3) limited-stage SCLC; (4) treatment without immunotherapy; (5) those whose irradiated sites did not include the brain, bone or lung; (6) radiotherapy before or after immunotherapy. Stages of these patients were defined based on the American Joint Committee of Cancer eighth edition TNM classification and staging system [26]. The ethical certification of the data in this retrospective study was approved by the Clinical Research Ethics Committee of Shandong Cancer Hospital and the informed consent was waived by our Institutional Review Board because of the retrospective nature of the study.

### Treatment Characteristics

The first-line therapy was defined as no anti-tumor therapy prior to immunotherapy. Second-line treatment referred to therapy after the failure of first-line treatment. Similarly, third-line treatment refers to the situation where both first—and second-line treatments have failed. Immune checkpoint inhibitors including PD-1/PD-L1 were administered in patients every 3 weeks intravenously. Combined chemotherapy was primarily consisted of etoposide with platinum. Some patients were treated with VEGFR represented by bevacizumab and anlotinib, and several were treated with a combination of ICI, CHT, as well as VEGFR. Each patient underwent conventional fractionated radiotherapy (CFRT) or Stereotactic body radiotherapy (SBRT) during immunotherapy, 2.0–3.5 Gy per fraction, 10 to 30 fractions or 7.0 Gy per fraction, 8 fractions. Radiation was administered once daily, five fractions a week. The normalization of radiation plans was 95% of the plan tumor volume needs receiving 100% of the prescribed dose. Besides, doses of organs at risk (OARs) were limited within the safe range.

### Data Collection

Blood cell counts of patients including neutrophil, lymphocyte, monocyte, platelet were recorded at three time periods. Pre-inflammatory biomarkers (pre-IBs) was defined as the blood indicators during the first stage, from the first day of immunotherapy, to the beginning of radiotherapy. And medio-IBs refers to the blood indicators ranged from the first fraction of radiotherapy to the last one. From the end of radiotherapy, to 2 months after immunotherapy (before the next line of treatment) we recorded post-IBs. We calculated MLR, NLR, PLR and SII and the delta-IBs were calculated using the formula:

$$\mathrm{delta}-\mathrm{IBs}=\mathrm{medio}-\mathrm{IBs}/\mathrm{pre}-\mathrm{IBs}-1$$

The 6/9/12/24-month OS refers to 6/9/12/24 months after the first day of immunotherapy. After the treatment, all patients were followed at regular intervals of every 3–6 months.

### Evaluation of Therapeutics Efficacy

Patients underwent enhanced computed tomography scans, magnetic resonance imaging and Emission Computed Tomography every 3 cycles of ICIs, routinely evaluated by no less than two associated chief physicians in imaging and radiology departments respectively. The evaluation of tumor response was depended on the principles set forth by immune response criteria in solid tumor (iRECIST) during immunotherapy [27].

### Statistical Analysis

By convert the continuous variables into binary variables, firstly we employed Spearman rank correlation test to figure out which blood index correlated with irradiated site. We used Kruskal–Wallis rank sum test, and then compared pairwise with a corrected α, whose significance was assumed at less than 0.0167 (0.05/3) to figure out the difference within group. Univariate logistic regression analysis was used to evaluate whether these clinical factors and blood indicators were correlated with OS. P-value of factor less than 0.05 was chosen into multivariate analysis. Finally, we analyzed the different OS rates in different treatment stages. IBM SPSS Statistics software (version 25.0, SPSS Inc., Chicago, IL), and RStudio, version 4.2.2 were used for statistical analysis.

## Results

### Patient Characteristics

A total of 45 patients (median age, 59 years old) with ES-SCLC who were treated with ICIs from July 2018 to October 2022 (follow-up cut-off) were enrolled in this study. Thirty-two patients (71.1%) had a history of smoking. Regarding the treatment stage, 12 patients (26.7%) were undergoing first-line therapy, 28 patients (62.2%) had received one prior therapy, and five patients (11.1%) had been treated with no less than two lines of therapy. The patients were divided into three groups according to the different irradiated metastases, including the brain, bone, and lungs, with or without lymphatic drainage. The baseline characteristics of the patients are summarized in Table 1. The delta-MLR in one patient and delta-EOS in three patients were lost.

**Table 1:** Clinical Baseline Characteristics

**All patients, *n* = 45**	**No. (%)**			
Sex	Male	Female		
	31 (68.9)	14 (31.1)		
Age (years)	≤ 60	> 60	Median	
	26 (57.8)	19 (42.2)	59	
ECOG performance status	≤ 1	≥ 2		
	32(71.1)	13 (28.9)		
BMI	Median (range)			
	24.3 (17.3–31.8)			
Smoking history	Never	Current or former	unknown	
	9 (20.0)	32 (71.1)	4 (8.9)	
Treatment stage	First-line	Second-line	Third-line and more	
	12 (26.7)	28 (62.2)	5 (11.1)	
Immunotherapy mode	ICI	ICI + CT	ICI + VEGFR	ICI + CT + VEGFR
	10 (22.2)	24 (53.3)	8 (17.8)	3 (6.7)
Irradiated site	Brain	Bone	Lung (drainage area lymph node)	
	22 (48.9)	13 (28.9)	10 (22.2)	
RT modality	CFRT	SBRT		
	*44 (97.8)*	*1 (2.2)*		

*Abbreviations*: *No.* Number, *ECOG* Eastern Cooperative Oncology Group, *BMI* Body Mass Index, *ICI* Immune Checkpoint Inhibitor, *CT* Chemotherapy, *VEGFR* Vascular Endothelial Growth Factor Receptor, *RT* Radiotherapy, *CFRT* Conventional Fractionated Radiotherapy, *SBRT* Stereotactic Body Radiotherapy

ICIs such as nivolumab, atezolizumab, durvalumab, camrelizumab, sintilimab, and islelizumab were administered intravenously every 3 weeks (Q3W) at an appropriate dose according to the height and weight of the patients. Chemotherapy plus platinum-etoposide was used as the first-line treatment, whereas the second-line and subsequent treatments were platinum-based albumin paclitaxel, bevacizumab, and anlotinib. After 4–6 cycles, ICI monotherapy was continued until tumor progression.

### Correlation Between Irradiated Sites and Blood Indicators

**Fig. 2:**
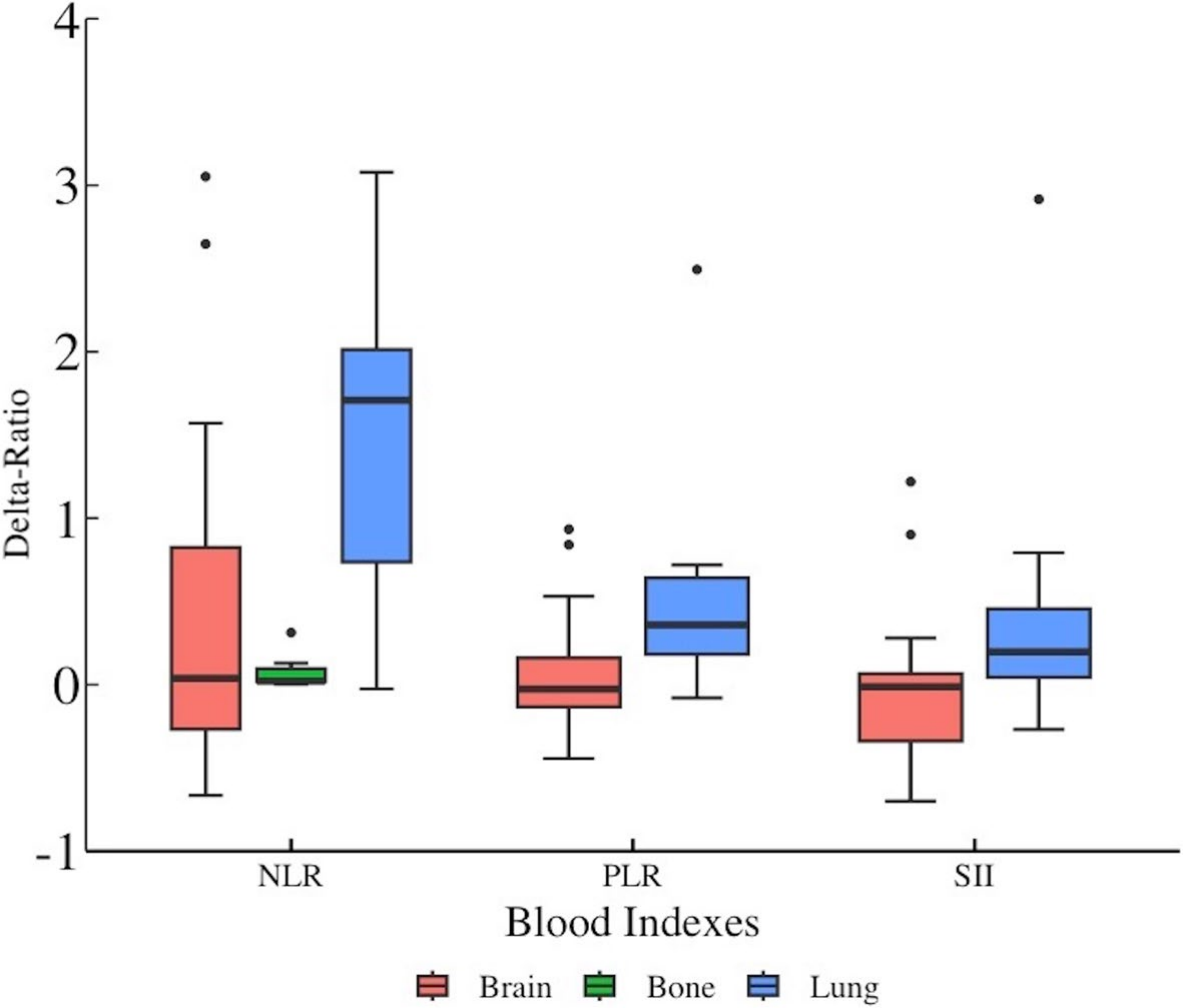
Three Out of Seven Positively Related Indicators were Selected. The Mean Ranks of Delta-NLR/PLR/SII were All the Lowest in the Brain Radiation Group During Immunotherapy

Three of the seven related indicators with p-values higher than 0.3 were selected in the Spearman rank correlation test. After the Kruskal–Wallis rank sum test, all three indicators were found to have significant inter-group differences (*p* < 0.05), namely delta-NLR, delta-PLR, and delta-SII (*p* = 0.004, *p* = 0.046, and *p* = 0.036, respectively). Pairwise comparisons were conducted among the groups for the three indicators. Eventually, statistically significant differences were found in the delta-NLR, delta-PLR, and delta-SII between the brain- and lung-irradiated groups (*p* = 0.004, *p* = 0.010, and *p* = 0.018, respectively), and in the delta-NLR ratio between the bone- and lung-irradiated groups (*p* = 0.002) (Table 2). The mean ranks were the lowest in the brain irradiated group for delta-NLR, delta-PLR, and delta-SII (mean rank = 19.50, 20.32, and 17.91, respectively) (Fig. 2).

**Table 2:** Correlation between Blood Indexes and RT Groups

Blood indexes	Correlation coefficient with group (*p*-value)	Inter-group difference (*p*-value)	Pairwise comparison (*p*-value corrected)
Delta-MLR	0.229(0.136)		
**Delta-NLR**	**0.392(0.008)**	**0.004**	**Brain-Lung (0.004)** **Bone-Lung (0.002)** Brain-Bone (0.811)
**Delta-PLR**	**0.374(0.011)**	**0.046**	**Brain-Lung (0.010)** Bone-Lung (0.577) Brain-Bone (0.183)
**Delta-SII**	**0.381(0.010)**	**0.036**	**Brain-Lung(0.018)** Bone-Lung (0.852) Brain-Bone (0.065)
Delta-L	- 0.168(0.288)		
Delta-M	0.152(0.299)		
Delta-EOS	0.251(0.110)		

*Abbreviation*s: *RT* Radiotherapy, *MLR* Monocyte-to-Lymphocyte Ratio, *NLR* Neutrophil-to-Lymphocyte Ratio, *PLR* Platelet-to-Lymphocyte Ratio, *SII* Systemic Immune-Inflammation Index, *L* Lymphocyte, *M* Monocyte, *EOS* Eosinophils

### Prognostic Factors and Survival Outcomes

**Fig. 3:**
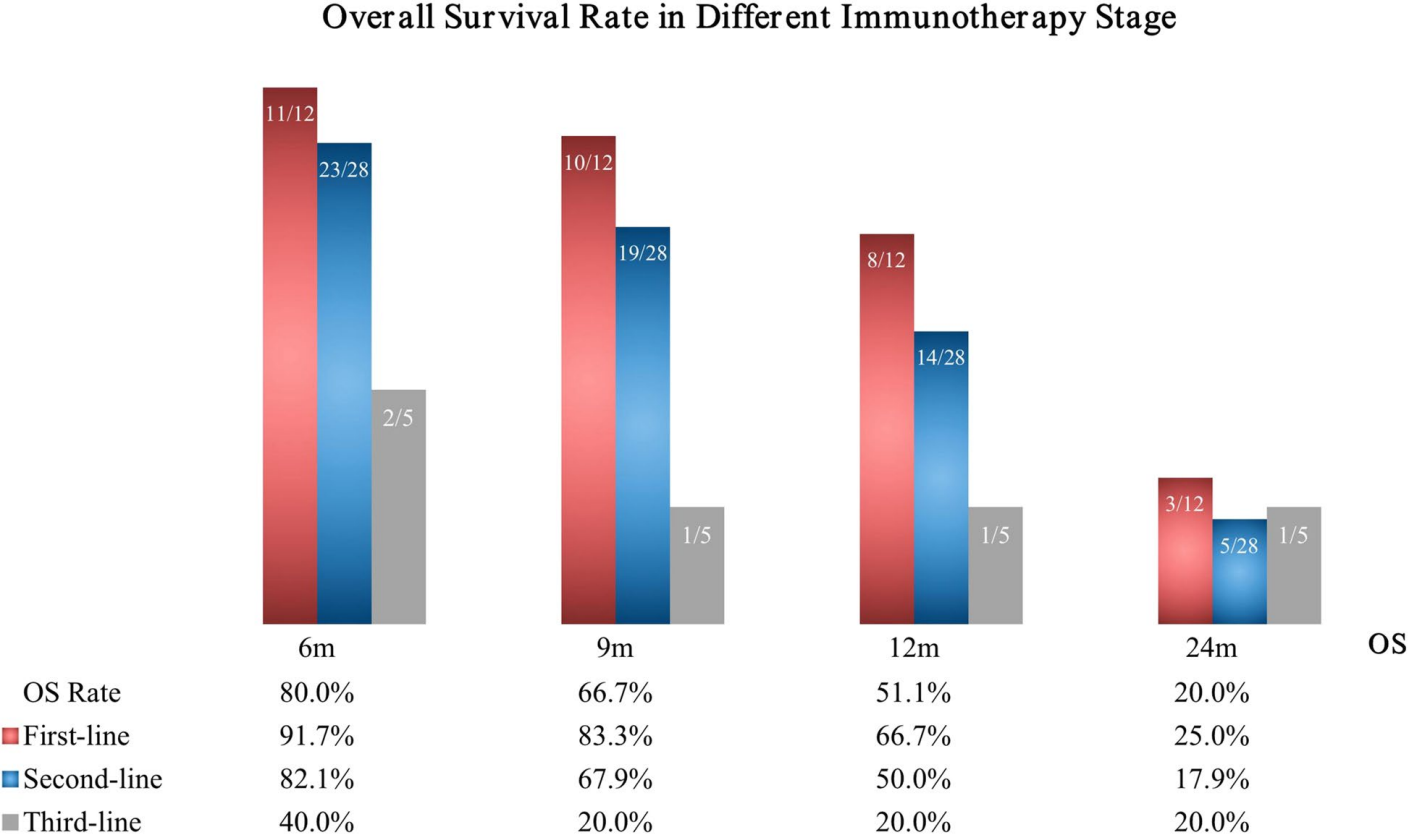
Datasets on Survival were Available for 40 Patients

As shown in the univariate and multivariate binary logistic regression analyses, treatment stage was an independent prognostic factor associated with the 9-month OS (odds ratio [OR], 0.219; 95% confidence interval [CI], 0.057–0.842; *p* = 0.027) (Table 3). Sub-group univariate analyses showed that only first-line therapy was significantly associated with the 9-month OS (*p* = 0.038). Survival data was available for 40 patients (88.9%). The 6-, 9-, 12- and 24-month OS rates were 80.0%, 66.67%, 51.11%, and 20.0%, respectively. Specifically, the OS for patients who underwent first-line immunotherapy combined with RT were 91.7% (11/12), 83.3% (10/12), 66.7% (8/12), and 25% (3/12), the OS following second-line therapy were 82.1% (23/28), 67.9% (19/28), 50.0% (14/28), and 17.9% (5/28), and the OS for patients who underwent third-line therapy were 40.0% (2/5), 20.0% (1/5), 20.0% (2/5), and 20.0% (1/5), respectively (Fig. 3).

**Table 3:** Univariate Analysis of Clinical Characteristics and Inflammatory Parameters in Correlation with 9 m OS

	**Univariate analysis** **(*n* = 45)**			**Multivariate regression** **(*n* = 45)**		
	***p* value**	**95% CI**	**OR**	***p* value**	**95% CI**	**OR**
**Clinical Characteristics**						
Age (≤ 60 years vs. > 60 years)	0.093	0.093–1.201	0.333			
Gender (Male vs. Female)	0.365	0.494–6.810	1.833			
BMI	0.226	0.925–1.391	1.134			
Smoking status (Never vs. With)	0.865	0.331–2.624	0.929			
ECOG performance status (≤ 1 vs. ≥ 2)	0.759	0.312–5.567	1.278			
Treatment stage 1st-line vs. 2nd vs. 3rd and more	0.033	0.069–0.892	0.248	0.027	0.057–0.842	0.219
ICIs modalities monotherapy vs. + CT vs. + VEGFR vs. three modes combined	0.305	0.310–1.443	0.669			
RT segmentation model (CFRT vs. SBRT)	1.000	-	-			
Groups (Irradiated sites)	0.432	0.618–3.088	1.381			
**Inflammatory Parameters**						
Delta-MLR	0.801	0.710–1.558	1.052			
Delta-NLR	0.456	0.661–2.519	1.290			
Delta-PLR	0.768	0.268–2.643	0.842			
Delta-SII	0.774	0.447–2.953	1.148			
Delta-L	0.306	0.353–27.638	3.125			
Delta-M	0.657	0.702–1.752	1.109			
Delta-EOS	0.338	0.457–1.309	0.773			

*Abbreviations*: *OS* Overall Survival, *CI* Confidence Interval, *OR* Odds Ratio, *BMI* Body Mass Index, *ICIs* Immune Checkpoint Inhibitors, *VEGFR* Vascular Endothelial Growth Factor Receptor, *CT* Chemotherapy, *CFRT* Conventional Fractionated radiotherapy, *SBRT* Stereotactic Body Radiotherapy, *ECOG* Eastern Cooperative Oncology Group, *MLR* Monocyte-to-Lymphocyte Ratio, *NLR* Neutrophil-to-Lymphocyte Ratio, *PLR* Platelet-to-Lymphocyte Ratio, *SII* Systemic Immune-Inflammation Index, *L* Lymphocyte, *M* Monocyte, *EOS* Eosinophils

## Discussion

We performed a retrospective analysis to assess the effects of radiation during immunotherapy on various organs in patients with multiple metastatic SCLC. Our objective was to investigate the selection process and the difference of immune activation effects across irradiated sites, as well as the survival outcomes associated with different stages of RIT in a real-world cohort.

Among the selected blood indices, namely NLR, PLR, and SII, we observed correlations with different irradiated organs. Notably, the mean ranks of delta-NLR, delta-PLR, and delta-SII were consistently lower in the brain-irradiated group during immunotherapy. Previous research identified these three indicators as prognostic markers of poor patient outcomes [28]. Hence, we can infer that RT to brain has the strongest activation effect and therapeutic response in ES-SCLC during immunotherapy. Furthermore, our analysis of prognostic factors for 9-month OS and survival rates across different treatment stages revealed that first-line immunotherapy combined with RT yielded a more favorable curative effect.

### Brain RT Improves the Immune Microenvironment Indicated by IBs

Gene expression profiling of SCLC patients has provided valuable insights into its heterogeneity, leading to its classification into four major subtypes: SCLC-A, SCLC-N, SCLC-P, and SCLC-Y. Most of these are immune-desert phenotypes characterized by a deficiency of CD8^+^T cells and an accumulation of regulatory T (Treg) cells and myeloid-derived suppressor cells (MDSCs) within the tumor microenvironment (TME). Consequently, the curative effect of immunotherapy alone is limited [24, 29]. A previous study showed that poly (ADP-ribose) polymerase inhibitors (PARPi) combined with RT reshaped the immune microenvironment of SCLC [30]. ICIs sensitize tumor cells to RT by normalizing the tumor vasculature and reducing hypoxia, thereby ameliorating the immune-desert TME [31]. Regardless of whether the patient undergoes prophylactic cranial irradiation (PCI), SRS, or WBRT, brain RT consistently plays a crucial role in systemic therapy strategies [32, 33]. Therefore, RT to the brain, lungs, or bones has a significant effect on immune activation during immunotherapy in patients with ES-SCLC.

Elevated NLR, PLR, and SII values from baseline are associated with shorter OS and progression-free survival [15, 34]. Consistent with these findings, a previous study suggested that lymphocytopenia is an unfavorable factor affecting immunotherapy outcomes in NSCLC patients [35]. This highlights the importance of improving the immune status by increasing the lymphocyte count, followed by a reduction in the above-mentioned ratios. In our study, among the brain-, bone-, and lung-irradiated groups, the median delta-ratios in the brain RT group were consistently the lowest during immunotherapy, suggesting that irradiation to brain exerts the strongest systemic activation effect in patients with ES-SCLC. In line with our findings, in advanced NSCLC, RT to the brain has been shown to exhibit the best immune activation effect and patient outcomes compared with other organs [36]. However, we did not find a significant correlation between the different irradiated groups and patient survival in ES-SCLC.

### PCI is Recommended for ES-SCLC During First-Line Immunotherapy

Owing to the extensive capillary network in the lungs, a significant number of anastomotic branches exist among the pulmonary vessels, vertebral veins, and cerebral vessels [37], providing a direct route for cancer cells to retrogradely enter the brain through the systemic circulation. However, in the case of other types of tumor cells, their initial destination is typically the lungs where they are transported via the venous system, and most cells are filtered through lung capillaries [38]. Consequently, the incidence of brain metastases (BMs) is substantially higher in SCLC. Compared with NSCLC, small cell carcinoma is more invasive and has a higher probability of intracranial metastasis, and approximately 60%–80% of patients who survive for more than 2 years develop brain metastases.

It is evident that RT to the brain not only activates the systemic immune state but also reduces the risk of brain metastasis. PCI is associated with a decrease in brain metastases in ES-SCLC, without a demonstrable impact on OS [39]. However, it should be noted that this reduction is primarily observed when RT is administered sequentially after chemotherapy.

Immunotherapy is very different from CHT; in particular, its combination with PCI to improve the TME is much stronger than chemotherapy alone. Consequently, in patients with ES-SCLC undergoing first-line systemic immunotherapy, PCI is recommended as the preferred approach over regular magnetic resonance imaging for monitoring intracranial metastases. Nevertheless, the absence of a significant correlation between different irradiation groups and patient survival in our study may be attributed to the limited number of cases examined. Further fundamental and prospective investigations are warranted to provide more robust evidence and elucidate the underlying reasons for these outcomes.

### Earlier Initiation of RIT in ES-SCLC Leads to Improved Survival Outcomes

The unique characteristics of the immunotherapy survival curve, known as the "late separation" and "long tail" effects, suggest that OS milestones such as 9- or 12-month OS rates may be associated with 5-year survival benefits [40]. In our analysis of factors associated with the 9-month OS, treatment stage was an independent prognostic factor, and subgroup analyses revealed a statistically significant correlation only between the first-line therapy and OS.

With the publication of results from the IMpower133, CASPIAN, ASTRUM-005, and CAPSTONE-1 studies, ICIs have been shown to greatly improve the survival of patients with ES-SCLC [41]. Although immunotherapy combined with chemotherapy (ICT) plays a significant role in ES-SCLC, data related to the combined efficacy and safety of RT is insufficient, and the results are controversial. A single-center retrospective study analyzing the safety and efficacy of first-line ICT combined with CTRT for ES-SCLC showed estimated OS rates of 97.1%, 80.2%, and 53.3% at 6 months, 1 year, and 2 years, respectively, with controlled safety [42]. However, another study indicated that the combination of first-line anti-PD-L1 blockade with BRT did not confer a significant survival benefit in patients with ES-SCLC with BMs [43]. These results suggest that the addition of RT to ICT may yield different outcomes. However, in our real-world analysis, RT combined with immunotherapy showed a trend of survival improvement in first-line RIT regardless of the irradiated site, and patients who received RIT earlier achieved higher survival rates.

The underlying cause of the diminished efficacy of immunotherapy combined with RT in ES-SCLC when treatment initiation is delayed remains unclear. Prospective studies on NSCLC, such as the KEYNOTE-189 and KEYNOTE-407 trials, demonstrated that patients transferred to the immunotherapy group after chemotherapy failure experienced significant improvements in survival outcomes [44, 45]. These findings suggest that earlier intervention leads to better treatment efficacy. However, studies focusing on SCLC are lacking. Although CHT eliminates tumor cells using cytotoxic drugs, it can induce an inflammatory response and promote the expression of drug-resistant tumor cells and factors that facilitate tumor metastasis [46].

Research has shown that a decrease in interleukin (IL)-2, IL-12, and interferon-γ coupled with an increase in lL-6, IL-8, IL-10, and tumor necrosis factor, can lead to the expansion of regulatory immune cells, such as Tregs, MDSCs, and M2 macrophages [47]. The accumulation of these regulatory immune cells elevates the expression of PD-1/CTLA-4, inhibits T-cell proliferation, and impairs the cytotoxicity of natural killer cells, resulting in immunosuppression [48]. Patients with SCLC typically exhibit weakened immune responses and stronger immunosuppression. Therefore, it is postulated that following prior chemotherapy, the body's immune system may be partially damaged, and immune cells within the TME may not be abundant. Consequently, in a heavily compromised immune system, immunotherapy combined with local RT may no longer achieve maximum effectiveness. In this real-world analysis, the OS rates of first-line immunotherapy combined with RT demonstrated a significant improvement compared to those of second- or third-line and subsequent therapies. Therefore, for patients with a favorable immune status, early administration of RIT is likely to yield enhanced local control and an extended estimated survival duration.

Therefore, considering the impact of radiation on the TME, we suggest that the integration of RT should be considered earlier in the era of immunotherapy for ES-SCLC.

## Conclusion

This study represents the first analysis of the different activation effects of RT on the brain, bones, and lungs with draining lymph nodes in 45 patients with ES-SCLC undergoing immunotherapy. The brain RT group showed the lowest mean rank of delta-NLR/PLR/SII, indicating that irradiation of the brain may have a more potent immune activation effect than that of other organs. Furthermore, ES-SCLC patients who received RIT earlier had a higher survival rate. Nevertheless, the underlying reasons for these findings remain unclear and there are ongoing clinical challenges pertaining to selecting the appropriate RT modality and identifying the optimal timing for RT intervention. Further investigations are required to overcome these challenges.

## Data Availability

The data supporting this study’s findings are available in the manuscript and the additional files or by request to corresponding authors.
